# Outdoor Air Pollution and Cardiovascular Diseases in Lebanon: A Case-Control Study

**DOI:** 10.1155/2015/810846

**Published:** 2015-01-13

**Authors:** Zeina Nasser, Pascale Salameh, Habib Dakik, Elias Elias, Linda Abou Abbas, Alain Levêque

**Affiliations:** ^1^Clinical and Epidemiological Research Laboratory (LCER), Doctoral School of Sciences and Technology, Lebanese University, Beirut 6573-14, Lebanon; ^2^School of Public Health, Free University of Brussels, Route de Lennik 808, CP 596, 1070 Brussels, Belgium; ^3^Clinical and Epidemiological Research Laboratory (LCER), Faculty of Pharmacy, Lebanese University, Beirut 6573-14, Lebanon; ^4^Division of Cardiology, Department of Internal Medicine, American University of Beirut, Riad El-Solh, Beirut 1107 2020, Lebanon; ^5^Division of Neurosurgery, Department of Surgery, American University of Beirut, Riad El-Solh, Beirut 1107 2020, Lebanon; ^6^Research Center in Epidemiology, Biostatistics and Clinical Research, School of Public Health, Free University of Brussels, Route de Lennik 808, CP 596, 1070 Brussels, Belgium

## Abstract

Outdoor air pollution is increasingly considered as a serious threat for cardiovascular diseases (CVD). The aim of this study is to investigate the association between outdoor pollutants and cardiovascular diseases among adults in Lebanon and to examine the possible moderator effect of cigarette smoking status on this association. A multicenter case-control study was conducted between October 2011 and October 2012. Cases were hospitalized patients diagnosed with CVD by a cardiologist while the control group subjects were free of any cardiac diseases. Information on sociodemographic characteristics, tobacco consumption, self-rated global health, pollution exposure, and other risk factors was collected using a questionnaire. The results of the logistic regression revealed that living near busy highway (OR 5.04, 95% CI (4.44–12.85), *P* < 0.001) and close to local diesel generator (OR 4.76, 95% CI (2.07–10.91), *P* < 0.001) was significantly associated with CVD. The association between the CVD and exposure to outside pollutants differed by cigarette smoking status. A clear difference was noted between nonsmokers and current smokers OR 4.6, 95% CI (1.10–19.25) and OR 10.11, 95% CI (7.33–20.23), respectively. Forthcoming studies are needed to clarify the potential link between outdoor air pollution and cardiovascular diseases in Lebanon. Public health interventions must be implemented to reduce air pollution and to improve air quality.

## 1. Introduction

Outdoor air pollution, composed of complex mixtures including gases (e.g., carbon monoxide (CO), nitrogen dioxide (NO_2_), ozone (O_3_), sulfur dioxide (SO_2_), and particulate matter (PM)), is increasingly considered a new threat to the cardiac system [1–4]. It is manifested by endothelial dysfunction, hypertension, and increased thrombogenic and inflammatory state [5]. Researchers noticed as early as the 1920s and 1930s the impact of air pollution on human health: high rates of death events occurred in London and Belgium after stagnant weather conditions caused by a sharp increase in the concentration of air pollutants for many days [6]. Literature uncovered the fact that even shy amounts of exposure to pollutants can have deleterious effects on the health in terms of morbidity and mortality [2, 7]. Several epidemiological studies on air pollution and cardiovascular diseases (CVD) are from developed countries [2, 8, 9]; unfortunately, this has been less investigated in developing countries, although they have similar or higher pollutant concentrations [10].

Motor vehicle emissions are recognized as a major source of outdoor air pollution [11]. Pollutants include organic compounds, carbon monoxide, nitrogen-sulfur oxides, unburned hydrocarbons, and PM [12]. Recently, traffic related air pollution is considered a risk factor for myocardial infarction (MI) [13]. Living within vicinity of highways, up to a radius of 100 meters, was considered as another risk of acute myocardial events [14]. Moreover, certain types of professions are at higher risk for cardiac events, such as taxi or bus drivers [13], waste incinerator workers [15], smelters operatives [16], or chimney sweeps [17, 18]. On the other hand, road vehicles were identified as a major contributing factor to air pollution emissions in the Middle East region [19]. Lebanon, a small growing nation, endured fifteen years of civil war leaving the infrastructure in a wreck, leading to a constant long shortage hours of electricity and forcing the public to install their own local diesel generators. Moreover, public transportation is mostly nonexistent and old vehicles with poor maintenance are widely spread [20]. In fact, a recent study conducted by Saliba et al. which measured PMs (PM_10_ and PM_2.5_: PM with aerodynamic diameters less than 10 and 2.5 *μ*m, resp.) concentrations in three sites in the capital Beirut (postwar 2006 construction activities, urban area, and populated area) revealed that Lebanon suffered from high PM levels [21] exceeding the World Health Organization (WHO) recommended values (PM_10_ 20 *μ*g/m^3^; PM_2.5_ 10 *μ*g/m^3^) [22].

So far no study has specifically assessed the association between outdoor air pollution and cardiovascular diseases in our country. The specific aim of our paper is to investigate this association among adults in Lebanon and to examine the possible moderator effect of cigarette smoking.

## 2. Methods

### 2.1. Study Design and Population

It is a multicenter case-control study, conducted between October 2011 and October 2012, in six hospitals in Lebanon, comparing CVD cases to a control group. Cases were defined as patients aged 40 years or above, hospitalized for cardiovascular disease, diagnosed with ST/non-ST elevation, myocardial infarction, stable/unstable angina, or heart failure, confirmed by a cardiologist based on their clinical presentation and laboratory exams [23]. The control group consisted of any subject aged 40 years or above admitted to the same hospitals for reasons excluding diabetes, hypertension, dyslipidemia, respiratory problems, or cardiovascular diseases. All participants signed an informed consent before enrolment in our study. Only 3 cases and 7 controls refused to take part in our research.

### 2.2. Data Collection

The Institutional Review Board (IRB) of our university waived the need for an official approval due to the observational nature of our study. A face to face interview was completed by three independent investigators trained in a standardized manner filling out an anonymous questionnaire. Charting was performed for the sake of data completion. Participant's anonymity and confidentiality were respected. Variables included a spectrum of sociodemographic characteristics such as age, gender, urban or rural residence, marital status (single, married, divorced, or widowed), education (illiteracy or primary, complementary, secondary, and university levels), and income-per-family-member (IPFM). IPFM, a measure defined as the household monthly income of a family divided by number of its members, was then categorized into quartiles (low, medium low, medium high, and highest). Health and behavior habits questions were included. For instance, cases and controls were asked to evaluate their health condition: “How would you describe your current health status” on a 10-point scale. Smoking status was also assessed for cigarette and water pipe smoking separately. Due to the small number of previous cigarette smokers, this subgroup was combined with current smokers. Passive smoking at home and at work was also weighed. On the subject of pollution, the traffic exposure indicator was assessed by the following question: “Are you living near a busy highway (<100 meters, >100 meters)?” The diesel emission exposure indicator was evaluated by the following question: “Are you living close to local diesel generator”? Traditional cardiovascular risk factors (CVRFs) were also collected from the hospital charts: hypertension, triglyceride, HDL (high density lipoprotein concentration), LDL (low density lipoprotein concentration), family history of cardiovascular disease (CVD), and obesity according to Body Mass Index scale (BMI) “normal weight" (BMI 18.5–24.9 kg/m^2^), “overweight" (BMI 25.0–29.9 kg/m^2^), and “obese” (BMI ≥ 30 kg/m^2^) [24].

### 2.3. Sample Size Calculation

Sample size calculation was done with a type I error of 5% and a study power of 80%. In the absence of baseline data, the exposure of healthy Lebanese residents to pollution was considered to be equal to 50%. The minimal sample size necessary to show a twofold increase in risk of cardiovascular diseases consisted of 330 subjects, divided into 111 cases and 222 controls. In the present study the simple size was 340 divided into 121 cases and 219 controls.

### 2.4. Statistical Analysis

The data entry was performed by independent laypersons that were unaware of the objectives of the study. Data cleaning was carried out by the researchers. Statistical analysis was performed using SPSS IBM version 20.0. Means with their 95% confidence intervals, medians with their interquartile ratio, and percentages were used to describe continuous and categorical variables. Statistical bivariate analysis was performed. The Pearson chi-square (*χ* ^2^) test was used for categorical variables. Student's *t*-test and Mann-Whitney *U* test were run for the continuous variables to compare their means. A *P* value < 0.05 was considered statistically significant. A multivariate analysis using logistic regression was carried out with CVD as the dependent variable. Adjusted odds ratios and their 95% confidence intervals were reported. The final logistic regression model was reached after ensuring the adequacy of our data using the Hosmer-Lemeshow test. Furthermore, due to the presumed relationship of cigarette smoking with both CVD [25] and exposure to outdoor air pollution [26], the interaction effect of this variable was tested (*P* value < 0.001). The regression model was stratified according to cigarette smoking status.

## 3. Results

### 3.1. Characteristics of the Study Sample

The baseline characteristics of our participants are described in Table 1. A total of 340 individuals were enrolled in the study: 219 (64.4%) controls and 121 (35.6%) cases. Living in urban areas, older age, and lower educational level were more common in cases than controls. Additionally, the BMI and the means of the risk factors (hypertension, triglycerides, and LDL) were significantly higher among cases.

### 3.2. Association between Smoking and CVD

The results of the bivariate analysis for smoking exposure (active or passive) and cardiovascular diseases are presented in Table 2. Current cigarette smokers had significantly higher risk of CVD than nonsmokers with an odds ratio (OR) of 1.92 and a 95% confidence interval (CI) between 1.22 and 3.01. A positive association was also found with passive smoking at home, OR 2.35 and 95% CI (1.46–3.78). Regarding water pipe and passive cigarette smoking at work, no significant association was pronounced.

### 3.3. Association between Outdoor Pollution, Cumulative Exposure, and CVD

All types of outdoor air pollution exposure were significantly associated with CVD. Furthermore, we noted an evidence of increased risk of CVD with long duration of living near a busy highway (*P* = 0.001, test for trend) and with extended duration of living close to local diesel generator (*P* < 0.001, test for trend) (Table 3).

### 3.4. Multivariate Analysis

All significant variables in bivariate analysis were included in the multivariate logistic regression. The model was suitable and Hosmer-Lemeshow test was adequate. Living near busy highway OR 5.04 and 95% CI (4.44–12.85) and living close to local diesel generator OR 4.76 and 95% CI (2.07–10.91) remained significantly associated with CVD after adjusting for HDL, LDL, triglyceride, SBP, and cigarette smoking status (Table 4). In an attempt to assess the dose-effect relationship, we also conducted another multivariate analysis where we treated the duration of living near busy highway and living close to local diesel generator as independent continuous variables instead of a dichotomous set. The association between cumulative exposure and CVD remained significant. We observed an increased occurrence of cardiovascular diseases with long exposure to outdoor pollutants. The OR was 1.05, 95% CI (1.02–1.07), and *P* = 0.016 for duration of living near busy highway and 1.38, 95% CI (1.10–1.78), and *P* = 0.014 for duration of living close to a local diesel generator (Table 4). An interaction effect of cigarette smoking was noticed on the association between CVD and living near busy highway (*P* for interaction: <0.001). Our results revealed a clear difference between nonsmokers and current smokers OR 4.6 and 95% CI (1.10–19.25) and OR 10.11 and 95% CI (7.33–20.23), respectively. However, no interaction was found while examining any possible effect of cigarette smoking status on the association between living close to diesel generator and CVD (*P* for interaction: 0.24) (Table 5).

## 4. Discussion

This is, to our knowledge, the first case-control study conducted to evaluate the association between outdoor air pollution and CVD among adults in Lebanon and to examine the potential moderator effect of cigarette smoking status on this association.

We found that CVD was associated with outdoor air pollution such as living near busy highway and living close to a local diesel generator. In accordance with our findings, several epidemiological studies showed that traffic particles are associated with cardiovascular diseases. A recent case-control study correlated long traffic exposure measured by PM_2.5_ and NO_2_ levels with an increase in acute myocardial infarction events [27]. Even more, evidence is emerging that long-term exposure to air pollution or traffic indicators is associated with measures of subclinical atherosclerosis [28, 29]. On the other hand, one of the risk factors proposed by other papers was the vicinity of the subjects' housing to major highways [30]. A five-year cohort study found that living close to traffic road was associated with mortality from cardiovascular incidence [31]. Our results also showed that cigarette smoking has a modification effect on the association, hence the importance of stratification by smokers status. The modifier effect of cigarette smoking status was evident in a study conducted by Pope III et al. [4]. Upon stratifying by cigarette smoking, we noticed that the risk of CVD of subjects exposed to outdoor air pollution is higher for current smokers compared to nonsmokers. A recent study evaluated the cellular toxicity of PM in the capital Beirut (near busy highways and urban background sites) using rat macrophage cells. It showed that PM stimulated the formation of reactive oxygen species (ROS) and triggered oxidative stress in these cells [32]. Numerous studies support evidence of a biological mechanism relating the air pollution toxins to changes in the cardiac system. In fact, PM exposure induced formation of atherosclerotic lesions in a study using rabbits as experimental specimens [33]. Regarding humans, a controlled exposure experiment to PM triggered both an acute and a long lasting inflammatory response [34, 35]. Toxic pollutants activate prothrombotic pathway and fibrinogen formation [28, 34], accelerating the formation of atherosclerosis, intensifying the risk of myocardial infarctions [2, 36]. In other studies, a predictor of cardiovascular events was assessed by elevation of C reactive protein level (a marker of myocardial infarction) upon exposure to PM [37, 38].

Consistent with other studies, dose-effect relationship, temporal relationship, and biological plausibility are strength points in our paper. Our study has however some limitations. The possibility of recall bias might be entertained due to the retrospective nature of our investigation. Cardiologist recruiting subjects were also unaware of their exposure status; this may decrease selection bias, which however cannot be excluded. There was also a possibility of interview bias since the questionnaires were filled by three independent investigators. Although we carried out a multivariate analysis to remove the confounding effects of several factors, there is still a possibility of residual confounding due to unmeasured factors. A further limitation in this study is that there are no national strategies in Lebanon aiming to assess the risk of air quality on the citizen's health. In addition, there are no actions in Lebanon to implement the indoor and outdoor Clean Air Act to control air pollution. Assessment of air pollution was based on self-reported information from both groups (cases and controls). The lack of air monitoring stations in Lebanon can represent inaccurate outdoor air pollutant levels. Therefore, the vicinity of sources was used as proxy instead of the quantitative air pollutant measurements. Studies conducted on measurement of air pollution concentrations are scarce and restricted only to the capital Beirut. Our paper should motivate the Lebanese government to establish permanent stations for monitoring general air quality across all the Lebanese territories.

In conclusion, we found that outdoor air pollution exposure, such as living near local diesel generator or a busy highway, was associated with CVD. These results were also confirmed by dose-effect and temporal relationship. Further studies should attempt to link air pollutants with the appropriate cardiac events (MI, heart rate variability, heart failure, unstable angina, etc.). In terms of public health practice, implementation of policy options to reduce traffic related air pollution and to develop a strategy to improve air quality is a necessity.

## Figures and Tables

**Table 1:** Baseline characteristics of the participants.

Characteristics	Controls *n* = 219 (%)	Cases *n* = 121 (%)	*P* value
Age			0.007^*^
40–44	43 (19.7)	7 (5.9)	
45–49	25 (11.5)	11 (9.2)	
50–54	16 (7.3)	18 (15.1)	
55–59	16 (7.3)	10 (8.4)	
60–64	28 (12.8)	20 (16.8)	
≥65	90 (41.3)	53 (44.5)	
Gender			0.902
Males	116 (53.0)	63 (52.5)	
Females	103 (47.0)	58 (47.5)	
Marital status			<0.001^*^
Single	36 (16.4)	1 (0.8)	
Married	157 (71.7)	104 (86.7)	
Divorced or widow	26 (11.9)	15 (12.5)	
Education			0.025^*^
Illiteracy	23 (10.5)	11 (9.7)	
Primary or less	82 (37.4)	60 (53.1)	
Complementary or less	48 (21.9)	25 (22.1)	
Secondary or less	37 (16.9)	10 (8.8)	
University degree	27 (13.2)	7 (6.2)	
Residency			0.033^*^
Rural	117 (53.4)	50 (41.3)	
Urban	102 (46.6)	71 (58.7)	
Income-per-family-member			0.221
Low	58 (27.0)	35 (30.2)	
Med. low	27 (12.6)	23 (19.8)	
Med. high	76 (35.3)	35 (30.2)	
Highest	54 (25.1)	23 (19.8)	
BMI^a^			0.004^*^
Normal (BMI 18.5–24.9)	71 (32.4)	22 (19.0)	
Overweight (BMI 25–29.9)	102 (46.4)	53 (45.7)	
Obese (BMI ≥30)	46 (21.0)	41 (35.3)	
Family history of CVD^b^			0.012^*^
No	143 (65.9)	62 (51.7)	
Yes	74 (34.1)	58 (48.3)	
Triglycerides mg/dL (mean ± SD)^c^	146.5 ± 43.6	202.2 ± 67.0	<0.001^*^
LDL mg/dL^d^ (mean ± SD)^c^	86.4 ± 46.3	128.9 ± 38.3	<0.001^*^
HDL mg/dL^e^ (mean ± SD)^c^	51.7 ± 15.6	43.3 ± 14.1	<0.001^*^
SBP mmHg^f^ (mean ± SD)^c^	122.5 ± 15.04	129.9 ± 19.1	<0.001^*^
Self-related global health (mean ± SD)^c^	8.3 ± 0.6	5.9 ± 1.4	<0.001^*^

^a^BMI, body mass index; ^b^cardiovascular disease; ^c^mean ± standard deviation; ^d^LDL, low density lipoprotein; ^e^HDL, high density lipoprotein; ^f^SBP, systolic blood pressure; ^*^ *P* value < 0.05 statistically significant.

**Table 2:** Subject's exposure to active and passive smoking and CVD.

Variable	Controls *n* = 219 (%)	Cases *n* = 121 (%)	OR (95% CI)
Cigarette smoking status			
Nonsmokers	137 (63.1)	57 (47.1)	
Current smokers	80 (36.9)	64 (52.9)	1.92 (1.22–3.01)
Passive cigarette smoking at home			
No	106 (51.2)	37 (30.8)	
Yes	101 (48.8)	83 (69.2)	2.35 (1.46–3.78)
Passive cigarette smoking at work			
No	21 (32.3)	15 (33.3)	
Yes	44 (67.7)	30 (66.7)	0.95 (0.42–2.14)
Water pipe smoking status			
Nonsmoker	170 (78.0)	100 (82.6)	
Current smoker	48 (22.0)	21 (17.4)	0.74 (0.42–1.31)

OR: odds ratio; CI: confidence interval.

**Table 3:** Exposure to outdoor air pollution and CVD.

Variable	Controls *n* = 219 (%)	Cases *n* = 121 (%)	OR (95% CI)
Highway proximity^a^			
>100 m	128 (58.4)	47 (38.8)	
<100 m	91 (41.6)	74 (61.2)	2.21 (1.40–3.48)
Living duration near highway^b^			
Never	143 (65.3)	53 (4.38)	1.00
1 to 14 years	18 (8.2)	11 (9.1)	1.14 (0.73–3.72)
15 to 30 years	26 (11.9)	28 (23.1)	1.51 (1.12–4.28)
31 years or more	32 (14.6)	29 (24.0)	2.28 (1.62–5.77)
Local diesel generator proximity^c^			
No	166 (75.8)	61 (50.4)	
Yes	53 (24.2)	60 (49.6)	3.08 (1.92–4.93)
Living duration near local diesel generator^d^			
Never	166 (75.8)	61 (50.4)	1.00
1 to 10 years	28 (12.8)	29 (24.0)	2.81 (1.55–5.11)
11 years and more	25 (11.4)	31 (25.6)	3.37 (1.84–6.16)

^a^Are you living near a busy highway?; ^b^duration of living near a busy highway; ^c^Are you living close to local diesel generator?; ^d^duration of living close to local diesel generator; OR: odds ratio; CI: confidence interval.

**Table 4:** Adjusted odds ratios with their 95% confidence intervals from the logistic regression of CVD among cases and control.

Global model	OR^a^	95% CI	*P* value
Living near a busy highway			<0.001^*^
<100 m	1.0		
>100 m	5.04	4.44–12.85	
Living close to local diesel generator	4.76	2.07–10.91	<0.001^*^
Dose-effect relationship^**^			
Duration of living near a busy highway	1.05	1.02–1.07	0.016^*^
Duration of living close to local diesel generator	1.38	1.10–1.78	0.014^*^

Global model: adjusted for triglyceride, HDL, LDL, SBP, and cigarette smoker status. OR^a^: adjusted odds ratio; CI: confidence interval; ^*^ *P* value < 0.05 statistically significant. ^**^Dose-effect relationship: adjusted for triglyceride, HDL, LDL, SBD, and cigarette smoker status. OR^a^: adjusted odds ratio; CI: confidence interval; ^*^ *P* value < 0.05 statistically significant.

**Table 5:** Adjusted odds ratios with their 95% confidence intervals from the logistic regression of CVD among cases and control stratified by cigarette smoking status.

Exposure type	Cigarette smoking					
	Nonsmokers			Current smokers		
	OR^a^	95% CI	*P* value	OR^a^	95% CI	*P* value
Living near a busy highway			0.030^*^			<0.001^*^
<100 m	1.0			1.0		
>100 m	4.60	1.10–19.25		10.11	7.33–20.23	
Living close to local diesel generator	4.97	1.64–15.08	0.005^*^	5.02	1.52–11.93	0.004^*^

Adjusted for triglyceride, HDL, LDL, and SBP; OR^a^: adjusted odds ratio; CI: confidence interval. ^*^ *P* value < 0.05 statistically significant.
